# Supplementary material for: Eco-Friendly Synthesis of PEtOz-PA: A Promising Polymer for the Formulation of Curcumin-Loaded Micelles
Source: Molecules. 2022 Jun 12;27(12):3788. doi: 10.3390/molecules27123788 (PMC9231041; doi:10.3390/molecules27123788)
Supplement: Supplementary file 1 [file molecules-27-03788-s001.zip › molecules-1763705-supplementary.pdf]

Eco-Friendly Synthesis of PEtOz-PA: A Promising Polymer for the Formulation of Curcumin-Loaded Micelles

Monica Nardi [1,2,*], Emiliana Sarubbi [1] and Satyanarayana Somavarapu [1]

1 Department of Pharmaceutics, UCL School of Pharmacy, London WC1N 1AX, UK; sarubbiemiliana@gmail.com (E.S.); s.somavarapu@ucl.ac.uk (S.S.)

2 Dipartimento di Scienze della Salute, Università Magna Græcia, Viale Europa, 88100 Germaneto, Italy

* Correspondence: monica.nardi@unicz.it; Tel.: +39-09613694116

Electronic Supplementary Material

Table of Contents

Experimental Section
General Information
Material
Synthesis of Poly(2-ethyl-2-oxazoline) palmitoyl ester (PEtOz-PA)
Figure S1: ^{13}C-NMR of PetOz-PA
Preparation of PEtOz-PA and PEtOz-PA(C) polymeric micelles.
Encapsulation efficiency (EE)
Figure S2: Calibration Curve for determination EE<br><br>**Calibration curve PetOz-PA(C) (1:20)**<br>**Calibration curve PetOz-PA(C) (1:10)**<br>**Calibration curve PetOz-PA(C) (1:5)**
In vitro release of curcumin
Figure S3: DPPH assay

Experimental Section

General information

Materials

Palmitoyl Chloride was purchased from Tokyo Chemical Industry (UK). Poly(2-ethyl-2-oxazoline) (PetOz) average M_n 10,000 and PDI ≤1.5, 2-Methyltetrahydrofuran (Me-THF), Curcumin (C, ≥94% curcuminoid content), pyrene (≥99%) and Erbium(III) trifluoromethanesulfonate were obtained from Sigma-Aldrich (UK). Deuterochloroform (99.8 atom % D) was purchased from Cambridge Isotope Lab. Inc. (USA). Acetonitrile, water (HPLC grade) and methanol were purchased from Fisher Scientific (United Kingdom). Deionized water was prepared in-house (PURELAB, ELGA, UK).

Synthesis of Poly(2-ethyl-2-oxazoline) palmitoyl ester (PEtOz-PA)

To a solution of Poly(2-ethyl-2-oxazoline) (10000 Mn; 0.1 mmol) were added a solution of palmitoyl chloride (0.15 mmol) in 2-MeTHF (8 mL) and 10 mol % of $Er(OTf)_3$ under stirring. The mixture was reacted for 4 h at reflux and was evaporated under vacuum. The mixture was poured in water saturated with $NaHCO_3$ and extracted with 2-MeTHF (3 × 10 mL). The organic layers were combined and dried on Na_2SO_4 and filtered, and the solvent was evaporated under vacuum. The purity of product obtained, was proved by Resonance Magnetic Spectrum.

Poly(2-ethyl-2-oxazoline) palmitoyl ester: Grey solid obtained in 90% yield; ^{1}H-NMR (500 MHz, $CDCl_3$): δ = 3.50–3.40 (m, 484 H, CH_2 alk), 2.45–2.40 (m, 134H, CH_2 alk), 2.20–2.30 (m, 102H, CH_2 alk, 2H, CH_2 palmitoyl), 1.60–1.80 (m, 42H, CH_2 alk), 1.25–1.35 (m, 10H, 10H, CH_2 palmitoyl), 1.20–1.25 (m, 16H, CH_2 palmitoyl), 1.10–1.20 (m, 343H, CH_2alk), 0.85–0.95 (m, 3H, CH_3 palmitoyl). ^{13}C-NMR (100 MHz, $CDCl_3$): 9.40, 15.29, 26.00, 29.00, 34.06, 43.21, 45.32, 66.10, 173.9, 174.2, 179.5. FT-IR (1743 cm^{-1} C=O stretching vibration).

Figure S1: ^{13}C-NMR PEtOz-PA

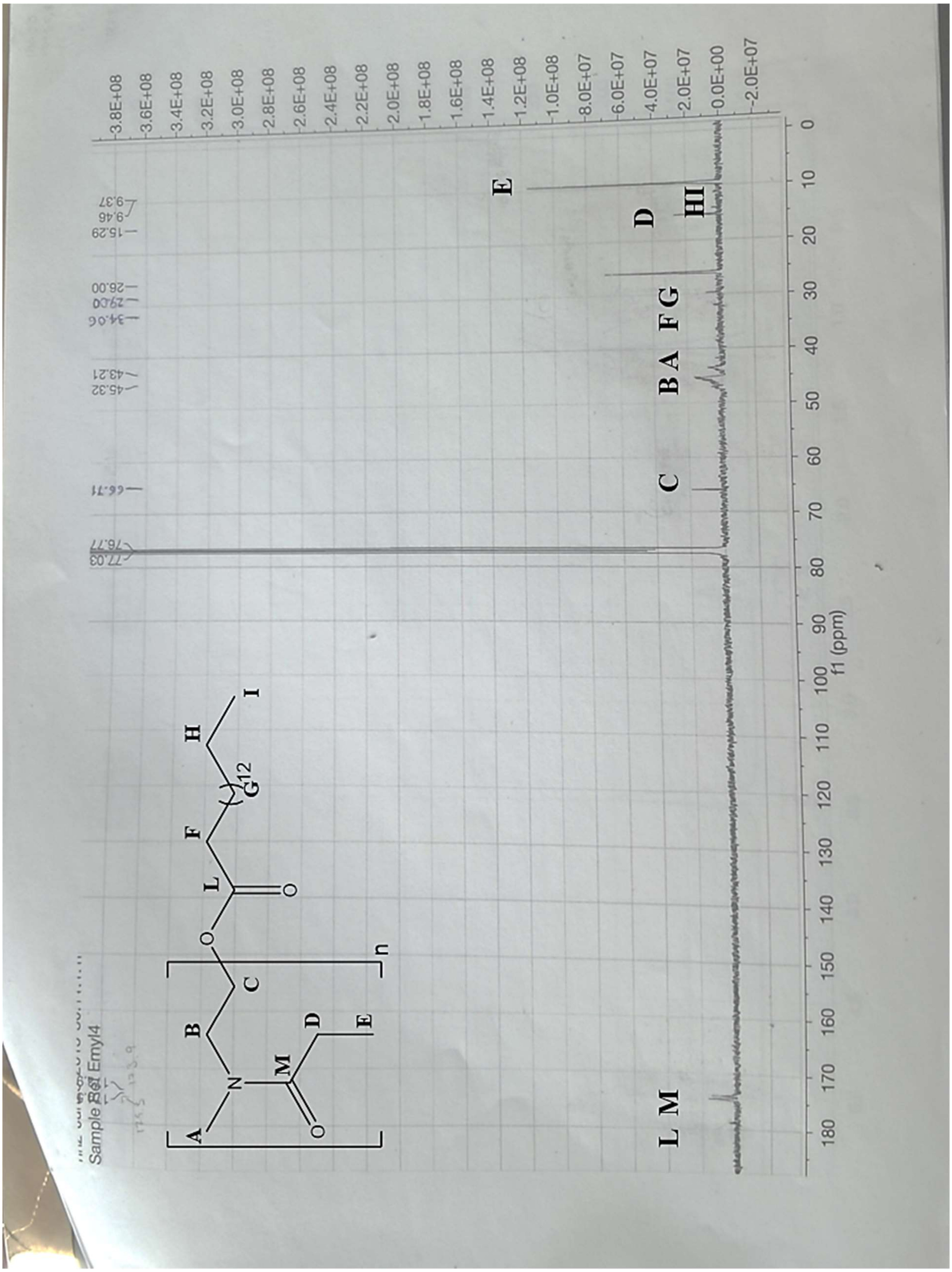

Preparation of PEtOz-PA and PEtOz-PA(C) polymeric micelles.

PetOz-PA(C) NPs were prepared using the double emulsion solvent evaporation technique as previously described [44]. The drug (C, 2 mg), PEtOz-PA (10, 20 and 40 mg) were dissolved in CH_2Cl_2 (6 ml) and the mixture sonicated using a VWR ultrasonic cleaner bath USC300T (VWR International Limited, UK) until complete dissolution. The solvent was then evaporated at 200 rpm and 80 ◦C under vacuum until a thin film was obtained using a rotary evaporator (Hei-VAP Advantage Rotary Evaporator, Heidolph, Germany). The resultant thin film was hydrated with 10 mL of warmed distilled water and sonicated for a further 15 min until the film was fully removed and dispersed in the water. The solution was filtered through a sterile 0.22 µm filter (30mm HPLC Syringe Filter Glass Fibre Prefilter/PTFE) and was stored at 4–8° C before being characterised. A proportion of the samples were lyophilized using a Virtis AdVantage 2.0 BenchTop freezedryer (SP Industries, UK) for further analysis.

Encapsulation efficiency (EE)

The formulations (1 ml) were diluted to 5 ml with methanol and, to evaluate curcumin content, were analyzed by reverse-phase HPLC on a Jasco LC-NetII/ADC, UV-2075 detector. A Luna Altech 4.6 × 150 mm Adsorbosphere C18 column with 5 μm particles of bonded silica gel with a guard column (4.6 × 7.4 mm Adsobosphere C18) was used. Absorbance chromatograms were obtained at 255 nm and all the measurements were performed at room temperature.

A binary mixture of acetonitrile/water (70/30, v/v) was applied as mobile phase with a flow rate of 1 mL/min and 20 µl injection loop. The method was validated for linearity, precision and recovery using standard solution (200µg/ml; 140µg/ml; 100µg/m; 70µg/ml; 50µg/ml; 35 µg/ml; 25 µg/ml). The calibration curve showed good linear regression (R^2 = 0.981) over the wide test ranges. The calibration curve was linear in the used solvent. The experiments were repeated for three times

Figure S2: Calibration Curve for determination EE

Calibration curve PetOz-PA(C) (1:20)

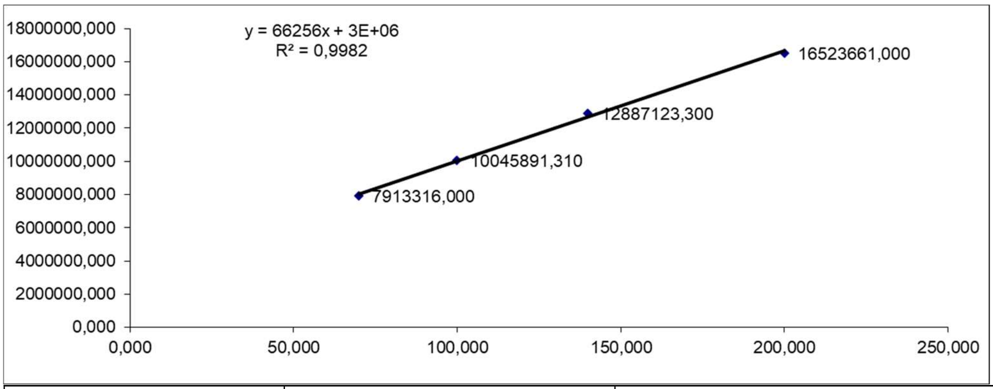

ug/ml	Area (nRIU*s)	
200,000	16523661,000	
140,000	12887123,300	
100,000	10045891,310	
70,000	7913316,000	
50,000	7701354,167	
35,000	5899447,000	
25,000	5060116,333	
**Sample 1**	5840020	83,61537068 ug/ml
**Sample 2**	5869100	84,05427433 ug/ml
**Sample 3**	5811011	83,17753864 ug/ml
		83,61573± 0,438368 ug/ml

Calibration curve PetOz-PA(C) (1:10)

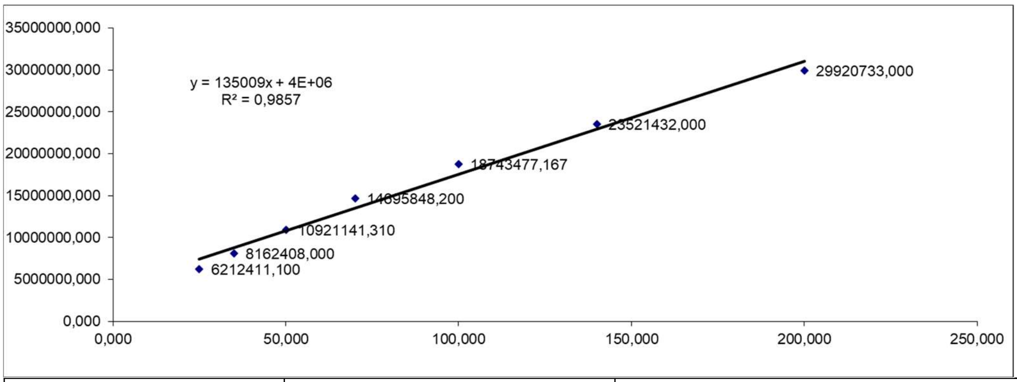

ug/ml	Area (nRIU*s)	
200,000	29920733,000	
140,000	23521432,000	
100,000	18743477,167	
70,000	14695848,200	
50,000	10921141,310	
35,000	8162408,000	
25,000	6212411,100	
**Sample 1**	13466001,67	96,77874561 ug/ml
**Sample 2**	13424321,89	96,47002709 ug/ml
**Sample 3**	13442898,98	96,60762601 ug/ml
		96,61879957 ± 0,154662267 ug/ml

Calibration curve PetOz-PA(C) (1:5)

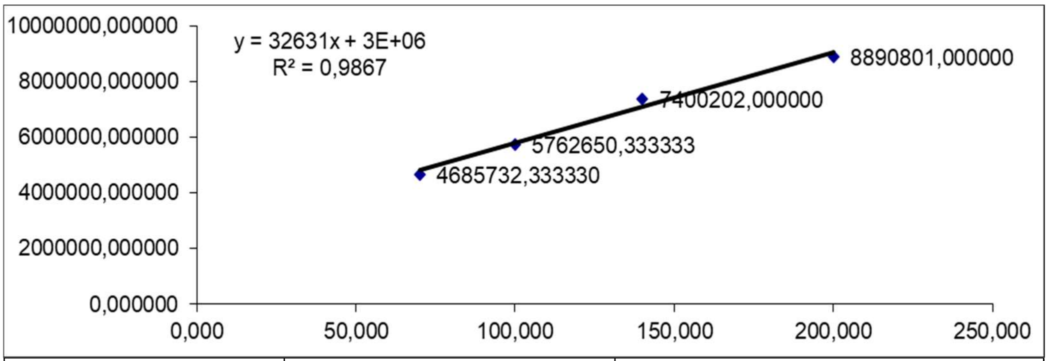

ug/ml	Area (nRIU*s)	
200,000	8890801,000000	
140,000	7400202,000000	
100,000	5762650,333333	
70,000	4685732,333330	
50,000	3349943,333	
35,000	2501573,333	
25,000	2002839,300000	
**Sample 1**		68,61389476 ug/ml
**Sample 2**		68,61757225 ug/ml
**Sample 3**		68,25282707 ug/ml
		68,49476469 ± 0,209532198 ug/ml

In vitro release of curcumin

The curcumin release was quantified in vitro by a membrane dialysis (MD) method [45], a widely used and versatile method for testing in vitro drug release of particulate formulations. The end-sealed dialysis bag (MW 4 kDa) containing the nanoparticules was placed in 30% ethanol in PBS (Phosphate buffered saline, 0.1 M, pH 7.4, 50 ml) with continuous magnetic stirring at 100 rpm. The buffer solution was used as dissolution medium at 37 ° C, and medium was collected at determined time point. The amount of drug released in the buffer solution was monitored by UV-vis spectroscopy, and the experiments were repeated for three times

DPPH assay

The DPPH radical-scavenging activity of the samples was assessed by the Gülçın method [47]. A 1,1-diphenyl-2-picrylhydrazyl radical solution was prepared by dissolving 0.0039 g of DPPH in MeOH to generate a 0.1 mM DPPH stock solution. Working solutions were prepared fresh daily by diluting the stock solution with solvent sufficiently to reduce the absorbance at 517 nm to 1.00 (±0.02). The DPPH radical solution (1 mL) was added to a solution (1 mL) of the compound to be tested in MeOH at different concentrations

Figure S3: DPPH Assay

PetOz-PA(C) (1:20)

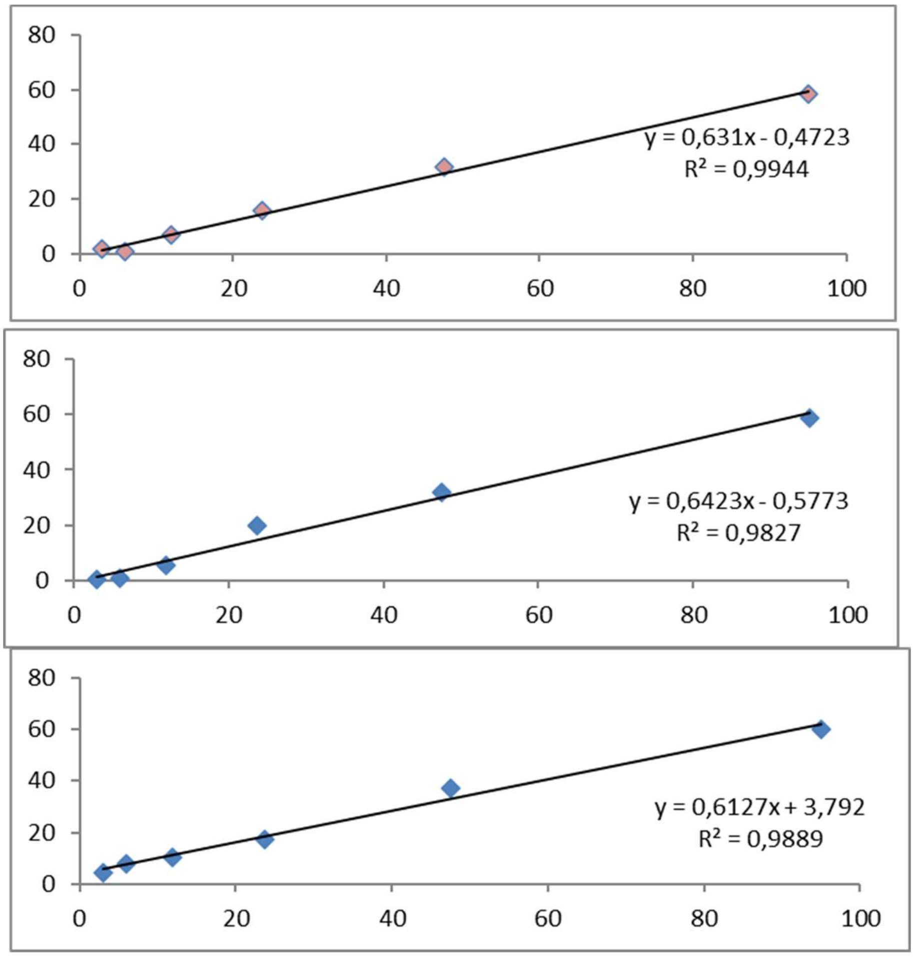

IC50	79,9878
IC50	78,74404
IC50	77,18545
**AVE**	**78,6391**
**SD**	**1,404117**

PetOz-PA(C) (1:10)

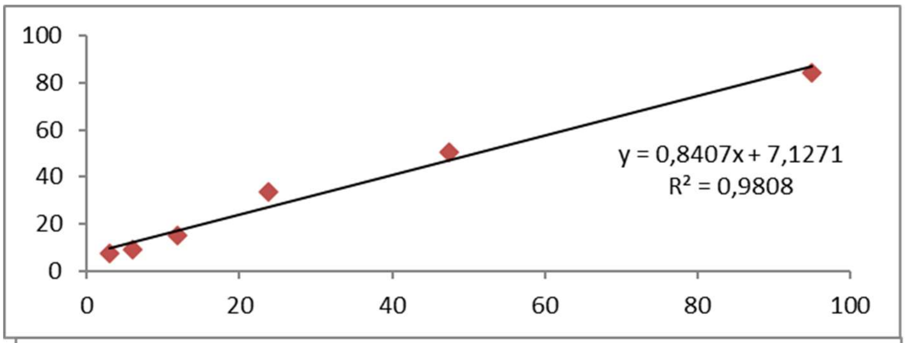

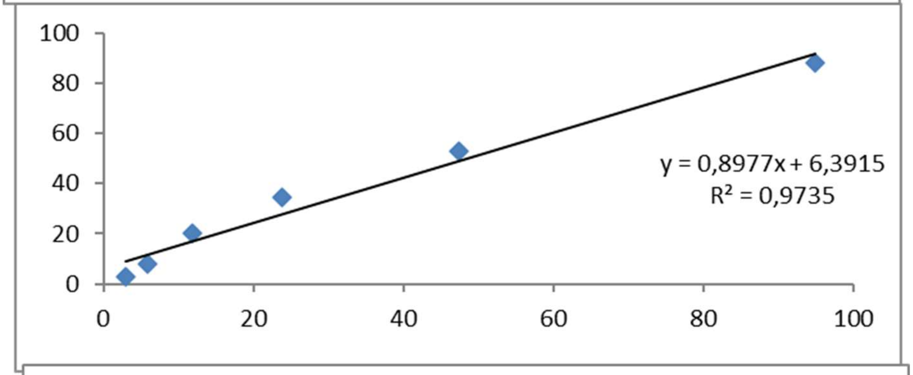

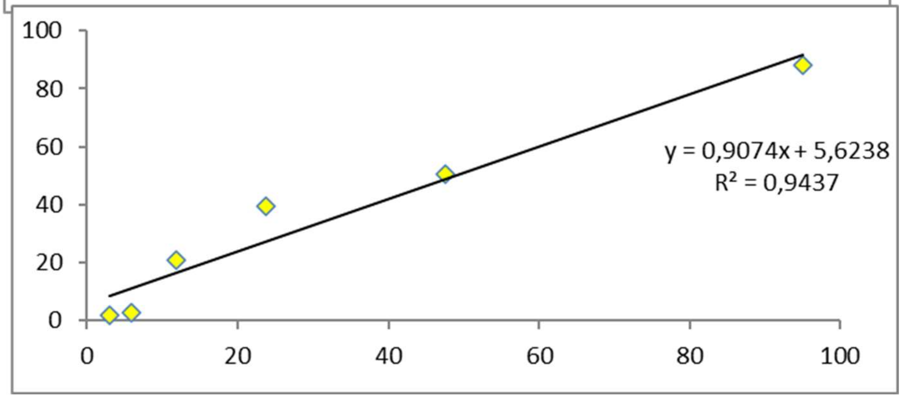

IC50	50,99667
IC50	48,57803
IC50	48,90478
**AVE**	**49,49316**
**SD**	**1,312285**

PetOz-PA(C) (1:5)

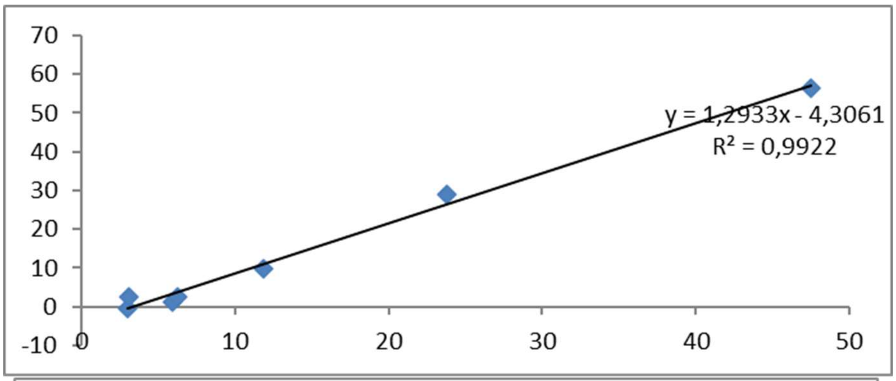

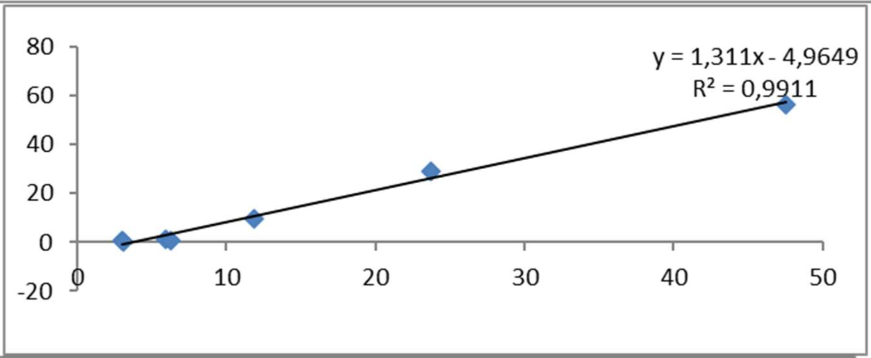

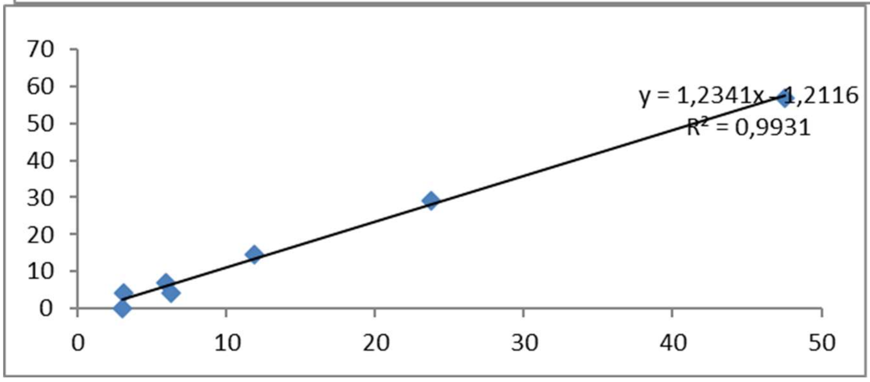

IC50	53,32962
IC50	41,92593
IC50	41,49712
**AVE**	**41,71153**
**SD**	**0,303215**

Curcumin

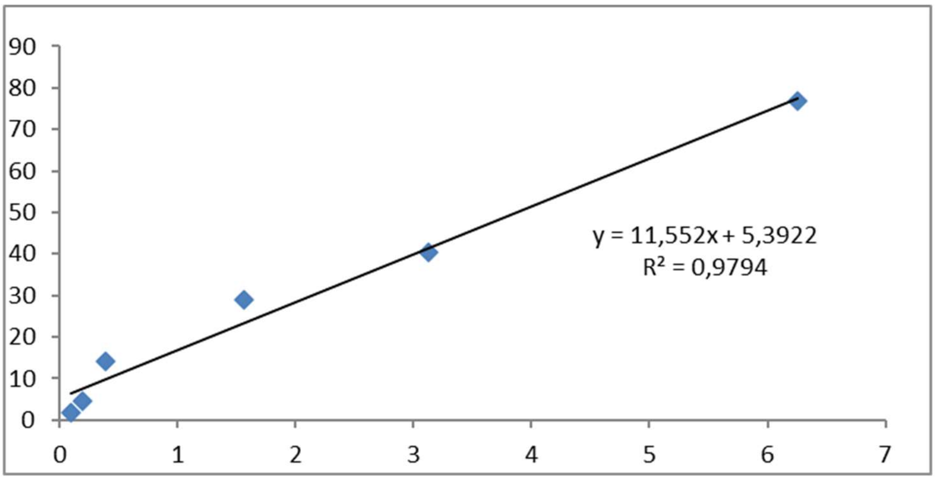

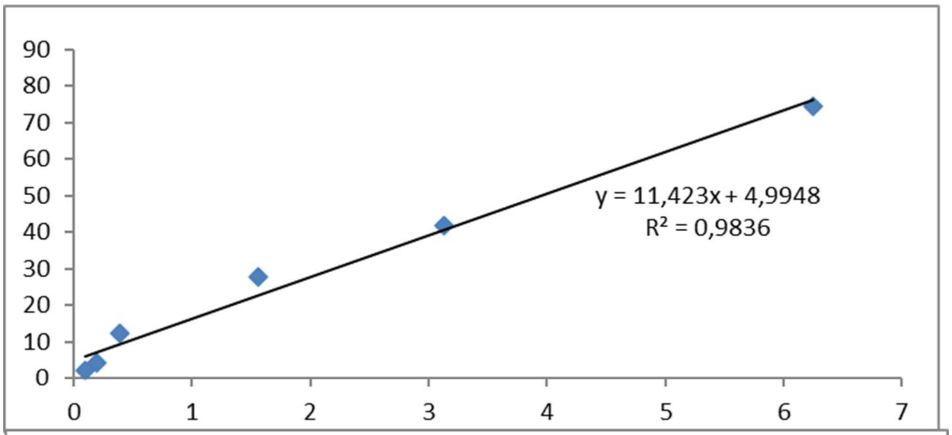

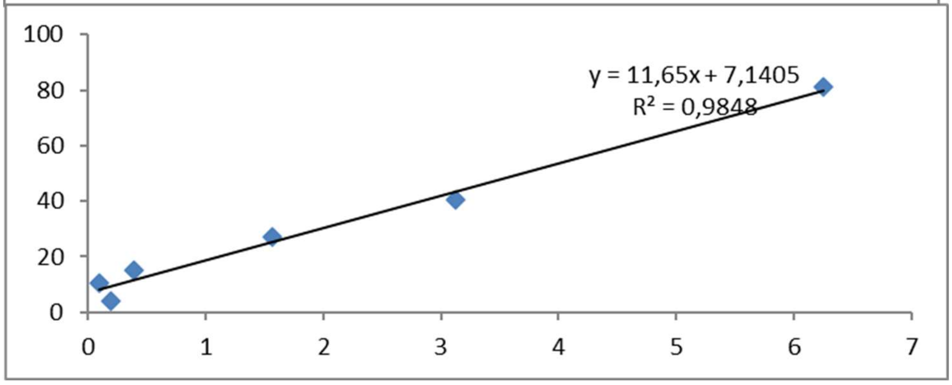

IC50	3,861479
IC50	3,939876
IC50	3,678927
**AVE**	**3,82676**
**SD**	**0,055435**

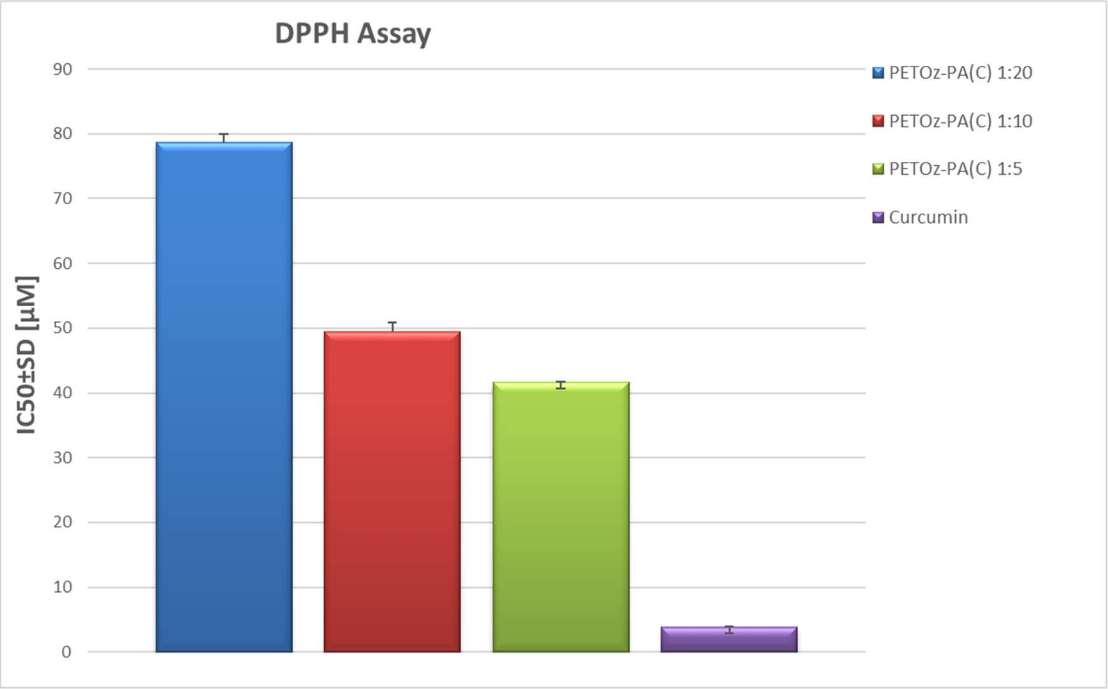
DPPH Assay
IC50±SD [µM]
90
80
70
60
50
40
30
20
10
0
PETOz-PA(C) 1:20
PETOz-PA(C) 1:10
PETOz-PA(C) 1:5
Curcumin
